# Corneal Confocal Microscopy Demonstrates Corneal Nerve Loss in Patients With Trigeminal Neuralgia

**DOI:** 10.3389/fneur.2020.00661

**Published:** 2020-07-24

**Authors:** John-Ih Lee, Theresa Böcking, Dagny Holle-Lee, Rayaz A. Malik, Bernd C. Kieseier, Hans-Peter Hartung, Rainer Guthoff, Christoph Kleinschnitz, Mark Stettner

**Affiliations:** ^1^Department of Neurology, Medical Faculty, Heinrich-Heine-University, Duesseldorf, Germany; ^2^Department of Neurology, University Medicine Essen, Essen, Germany; ^3^Weill Cornell Medicine-Qatar, Doha, Qatar; ^4^Division of Cardiovascular Medicine, University of Manchester, Manchester, United Kingdom; ^5^Department of Ophthalmology, Medical Faculty, Heinrich-Heine-University, Duesseldorf, Germany

**Keywords:** corneal confocal microscopy, trigeminal neuralgia, corneal nerve loss, headache, corneal nerve fiber

## Abstract

**Background:** The diagnosis of trigeminal neuralgia (TN) is challenging due to the lack of objective diagnostics. Corneal confocal microscopy (CCM) is a non-invasive ophthalmic imaging technique, which allows quantification of corneal nerve fibers arising from the trigeminal ganglion and may allow the assessment of neurodegeneration in TN.

**Methods:** CCM was undertaken in 11 patients with TN and 11 age-matched healthy controls. Corneal nerve fiber density (CNFD), corneal nerve branch density, corneal nerve fiber length (CNFL), corneal nerve fiber width, corneal nerve fiber area, and dendritic cell and non-dendritic cell density with or without nerve fiber contact were quantified.

**Results:** Patients with TN had significantly lower CNFD and CNFL but no difference for any other corneal nerve or dendritic cell parameter in the ipsilateral and the contralateral cornea compared to the control group. There was no significant difference in corneal nerve and cell parameters between patients with TN with and without involvement of the ophthalmic nerve (V1) or with nerve vessel conflict.

**Conclusion:** Corneal confocal microscopy is a rapid non-invasive imaging technique that identifies symmetrical corneal nerve loss in patients with TN.

## Introduction

Trigeminal neuralgia (TN) is rare, with incidence ranging from 4.3 to 27 new cases per 100,000 people per year (1–3), but can markedly impair activities of daily living (4) and, in severe cases, lead to suicide (5). TN is often misdiagnosed (6) as the diagnosis relies primarily on patient history, with no objective diagnostic or laboratory tests. Furthermore, the differential diagnosis between TN and other headache or odontogenic disorders can be challenging (7). There is an unmet need for objective methods to support the diagnosis of TN.

Corneal confocal microscopy (CCM) is a non-invasive ophthalmic imaging technique which can rapidly quantify corneal sensory nerve fibers arising from the trigeminal ganglion using automated image analysis (8, 9). It has good diagnostic validity in diabetic neuropathy (10, 11) and has identified axonal loss in chronic inflammatory demyelinating polyneuropathy (CIDP) (12), hereditary sensory and autonomic neuropathy (13), Charcot–Marie–Tooth disease type 1A (14), Fabry disease (15), and idiopathic small fiber neuropathy (16). Quantification of Langerhans cells into dendritic and non-dendritic cells may also provide insights into the immune alterations *in vivo* (17, 18).

Corneal nerve fiber density (CNFD) and corneal nerve fiber length (CNFL) are reduced in patients with chronic migraine (19), especially those with photophobia (20), although a recent study has demonstrated increased corneal nerve branch density and tortuosity and Langerhans cells in patients with episodic migraine (21). We have recently shown a reduction in corneal nerve fiber density and length and an increase in Langerhans cell density in patients with burning mouth syndrome (22). A previous study showed a symmetrical reduced corneal nerve fiber length in patients with TN of varying duration who had undergone microvascular or trigeminal ganglion decompression (23).

To our knowledge, CCM has not been undertaken in patients with active TN. The objective of this study was to assess if CCM can identify alterations in corneal nerve fibers and Langerhans cells in patients with TN.

## Patients and Methods

### Ethics

The local Ethics Committee of Heinrich Heine University Duesseldorf (Study-number 5431R) and of the University of Essen (Study-number 16-6929-B0) approved this prospective observational study. Written informed consent was obtained from all participants in accordance with the Declaration of Helsinki.

### Participants

Fifteen patients were prospectively recruited from the tertiary headache center in the Department of Neurology, University Hospital Essen. CCM was performed in the Department of Neurology, Heinrich Heine University Duesseldorf or in the Department of Neurology, University of Essen, Germany, between 2016 and 2018. The inclusion criterion was the diagnosis of TN according to the International Headache Society. TN patients suffered from classical (*n* = 6) or idiopathic (*n* = 5) TN. The pain attacks lasted from 1 s to 2 min. The frequency ranged from two times per day to >100 times per day. Six patients had purely paroxysmal TN with pain-free episodes between attacks in the affected trigeminal distribution and five patients had TN with concomitant continuous or near-continuous pain between attacks in the affected trigeminal distribution. All the patients fulfilled the TN criteria of ICHD-3; secondary TN patients were excluded. The exclusion criteria were secondary TN, any injury of the cornea, diabetes mellitus, and polyneuropathy. Two patients with diabetes mellitus and two patients with polyneuropathy were excluded. All 11 patients included in this study were pharmacologically treated with at least one anticonvulsant and had active TN. Three out of the 11 TN patients had received microvascular decompression (Janetta surgery) in the past.

Eleven age-matched healthy controls were recruited from the University of Manchester, United Kingdom (North Manchester Ethics Committee). The controls underwent blood workup and extensive neurological assessment and neurophysiology to exclude neuropathy.

### CCM Methodology

All the study participants were scanned using a CCM [Heidelberg Retinal Tomograph III Rostock Cornea Module (HRT III RCM); Heidelberg Engineering GmbH, Heidelberg, Germany], as described previously (9, 12). Using the section mode, several scans of the entire depth of the cornea were recorded by turning the fine focus of the objective lens backward and forward to focus on the sub-basal nerve plexus at the center of the cornea, and six high-quality images were analyzed (24).

To limit any bias, an independent investigator undertook fully automated image analysis to quantify corneal nerve fiber density (CNFD), corneal nerve branch density, corneal nerve fiber length (CNFL), corneal nerve fiber width, and corneal nerve fiber area using ACCMetrics software (ACCMetrics; M.A. Dabbah, Imaging Science and Biomedical Engineering, Manchester, UK), a tool developed alongside the manual image analysis system (9). Langerhans cells were analyzed manually from the same images used for nerve fiber analysis. Based on their morphology, the cells were classified as dendritic cells (DC) if the cell body had dendritic extensions and non-dendritic cells if there were no dendritic extensions, and they were further subclassified into those displaying contact (F) or no contact (P) with nerve fibers. For dendritic cells, nerve fiber contact was defined by direct contact of one or more of the dendrites or the cell body with the nerve fiber, and for non-dendritic cells, nerve fiber contact was defined by direct contact of the cell body with a nerve fiber.

### Statistical Evaluation

Statistical analyses were performed using SPSS Statistics 20 (IBM). Two-tailed Mann–Whitney *U* test was used to analyze the differences in CCM parameters between patients with TN and controls. Two-tailed non-parametric Wilcoxon matched pairs test was performed to test for differences in CCM parameters between the ipsilateral and the contralateral cornea of TN patients. Bonferroni correction for multiple testing was performed. Subjects with missing data were excluded from the respective analysis. *P*-values < 0.05 were considered as significant.

## Results

### Patients

Eleven patients, median age 58.43 [interquartile range (IQR) 48.63–62.43], with a median duration of TN of 5.97 (IQR 2.50–10.04) years and varying degrees of trigeminal nerve branch involvement with and without vessel conflict were compared to 11 age-matched healthy control subjects with median age of 58.53 (IQR 48.56–62.77) years (Table 1).

**Table 1:** Baseline parameters of the 11 trigeminal neuralgia (TN) patients, absolute numbers and percentages, or median with interquartile range (IQR) are provided for demographics.

	**TN (*n* = 11)**
Median age in years (IQR)	58.43 (48.63–62.43)
Male gender	4 (36%)
Right side affected	3 (27%)
Left side affected	8 (73%)
Both sides affected	0 (0%)
V1 affected	4 (36%)
V2 affected	11 (100%)
V3 affected	5 (45%)
Vessel nerve conflict	6 (55%)
Median duration of diagnosed TN in years (IQR)	5.97 (2.50–10.04)

### CCM

**Figure 1:**
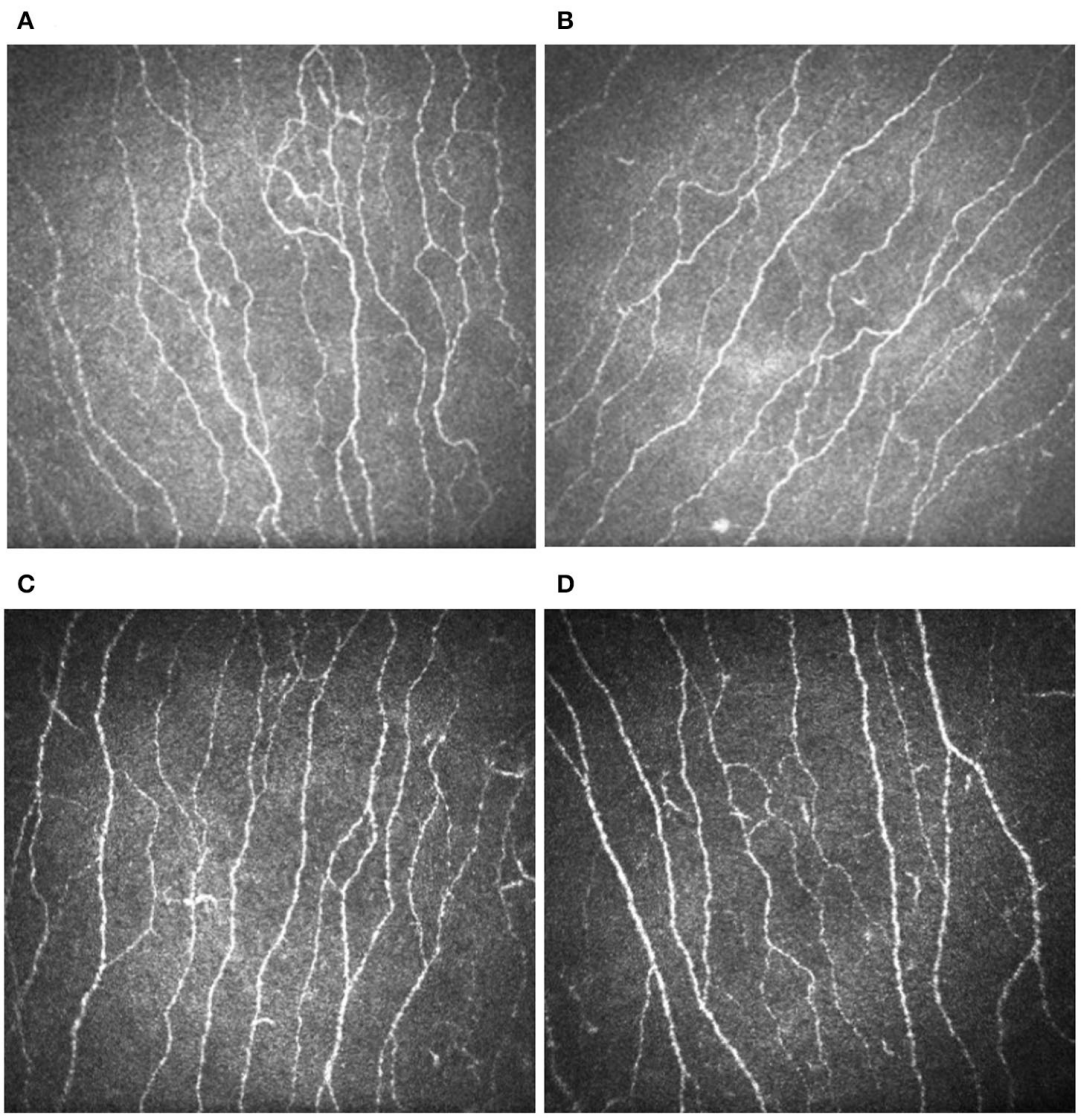
Corneal confocal microscopy images showing a reduction in the sub-basal nerve plexus (white lines) in the ipsilateral **(A)** and contralateral **(B)** cornea of a patient with trigeminal neuralgia and the left **(C)** and right **(D)** cornea of a healthy control.

CNFD and CNFL were significantly lower in the ipsilateral cornea of patients with TN compared to those in control subjects (Table 2, Figure 1). There was no difference in the DC density between the ipsilateral cornea of patients with TN and the control group (Table 2). Corneal nerve and dendritic cell parameters were comparable between the ipsilateral and the contralateral cornea (Table S1).

**Table 2:** Corneal confocal microscopy findings of controls compared to the ipsilateral, pain-affected side in trigeminal neuralgia patients are presented as mean, standard deviation, and *P*-value (*P*-value < 0.05 considered as statistically significant; n.s.—no significant difference).

	**Control (*n* = 11)**	**TN (*n* = 11)**	***P*-value**
Corneal nerve fiber density (no./mm^2^)	31.15 ± 4.27	22.41 ± 5.69	*P* < 0.01
Corneal nerve branch density (no./mm^2^)	41.23 ± 15.04	27.89 ± 19.08	n.s.
Corneal nerve fiber length (mm/mm^2^)	18.06 ± 2.45	13.61 ± 3.58	*P* < 0.05
Corneal total branch density (no./mm^2^)	56.94 ± 17.96	43.09 ± 29.76	n.s.
Corneal nerve fiber area (μm^2^)	6.07 ± 1.07	5.74 ± 2.12	n.s.
Corneal nerve fiber width (μm)	20.81 ± 1.22	19.80 ± 0.51	n.s.
Dendritic cells (DC; contact, F) (no./mm^2^)	5.26 ± 4.08	7.31 ± 7.56	n.s.
Non-dendritic cells (NC; F) (no./mm^2^)	3.34 ± 3.84	4.14 ± 3.22	n.s.
DC (no contact, P) (no./mm^2^)	5.79 ± 4.98	15.24 ± 14.01	n.s.
NC (P) (no./mm^2^)	15.01 ± 14.58	16.03 ± 17.31	n.s.

In a subgroup analysis, there was no difference for ipsilateral CCM parameters between TN patients with (*n* = 4) and without (*n* = 7) clinical involvement of the ophthalmic nerve (V1) (Table S2). The clinical involvement of V1 was based on the occurrence of pain attacks in the ophthalmic division of the trigeminal nerve, affecting the skin of the upper face and scalp. Furthermore, there was no difference for ipsilateral CCM parameters between TN patients with (*n* = 6) and without (*n* = 5) vessel nerve conflict on magnetic resonance imaging (Table S3).

## Discussion

TN is a rare headache disorder (1–3) which is often mis- or underdiagnosed (6) and can have devastating effects on the patients' quality of life (4). There is an unmet need for a fast and non-invasive method to support the diagnosis of TN. CCM is a fast, non-invasive, and reproducible method that has been used to quantify corneal nerve fibers to identify axonal loss in various peripheral neuropathies (10–16).

CNFD and CNFL are reduced in patients with chronic migraine (19), especially those with photophobic migraine (20). However, in a recent study of patients with episodic migraine, nerve branching and tortuosity and Langerhans cell density were increased compared to controls, which are indicative of nerve regeneration and inflammation (21). In a recent study of patients with burning mouth syndrome, corneal nerve fiber density and length were lower and Langerhans cell density was higher compared to control subjects (22).

To our knowledge, CCM has not been performed in patients with active TN. We have no clear explanation as to why corneal nerve fiber density and length are equally reduced in both the ipsilateral and the contralateral cornea of TN patients compared to controls. In this context, corneal nerve loss may be a marker for peripheral neurodegeneration in TN and does not provide an explanation for neuralgic pain. Indeed although the etiology of postherpetic neuralgia (PHN) and TN differ, Truini et al. (25) showed absent C-fiber-related laser evoked potentials and mild epidermal nerve fiber loss on the unaffected side of two out of 10 patients with PHN, supporting subclinical afferent pathway involvement in the unaffected side (26, 27).

Previously, in a study of 21 patients with TN and balloon compression of the trigeminal ganglion, there was evidence of corneal hypoesthesia and reduced corneal nerve fiber length in the ipsilateral and the contralateral cornea, while in those who had undergone microvascular decompression, there was no corneal hypoesthesia but there was a reduction in corneal nerve fiber length (23) compared to healthy controls (28). This indicates a more global impact on corneal nerves in TN, irrespective of operative procedures. Indeed in the present study we show that corneal nerve loss was comparable between TN patients with or without nerve vessel conflict or clinical involvement of V1.

A biopsy study of the nerve root in TN patients has shown zones of demyelination and axonal degeneration (29). Diffusion tensor imaging studies have revealed significantly lower fractional anisotropy values in the vulnerable zone of the trigeminal ganglion (30) and prolonged latencies and reduced amplitudes in the nociceptive blink reflex and pain-related evoked potentials (31) on the ipsilateral compared to the contralateral side.

We acknowledge the relatively small cohort of patients studied is a major limitation and could affect the reliability of our findings. Nevertheless, this is the first study to show corneal nerve loss in patients with ongoing TN. The corneal nerve loss demonstrated with CCM may reflect trigeminal nerve involvement in patients with TN, and it may serve as a non-invasive and objective marker of neurodegeneration to help diagnose TN. Larger, longitudinal studies of CCM in relation to disease severity and therapy are needed to confirm our findings in order to assess the utility of this technique as a surrogate marker in TN.

## Data Availability Statement

The datasets generated for this study are available on request to the corresponding author.

## Ethics Statement

The studies involving human participants were reviewed and approved by local Ethics Committee of Heinrich Heine University Duesseldorf and local Ethics Committee of the University of Essen. The patients/participants provided their written informed consent to participate in this study.

## Author Contributions

J-IL, DH-L, and MS were responsible for study design, data acquisition, data analysis, drafting of the manuscript, and revision of the manuscript for important intellectual content and approved the version to be published. TB was responsible for data acquisition, data analysis, drafting of the manuscript, and revision of the manuscript for important intellectual content and approved the version to be published. RM, BK, H-PH, RG, and CK revised the manuscript for important intellectual content and approved the version to be published. All authors contributed to the article and approved the submitted version.

## Conflict of Interest

J-IL has received honoraria for speaking/consultation from Bayer Healthcare, Boehringer Ingelheim, Ipsen, Allergan, Novartis, Teva, and Daiichi-Sankyo as well as travel grants from Bayer Healthcare, Merz Pharmaceuticals, Ipsen, and Allergan outside the submitted work. DH-L has received honoraria for research, talks, and advisory boards from Lilly, Hormosan, Allergan, Teva, Sanofi, and Novartis outside the submitted work. RM has received honoraria for educational lectures for Novo Nordisk, Pfizer, and Merck. H-PH has outside the work presented here, received fees for serving on steering or data monitoring committees from Bayer Healthcare, Biogen, Celgene Receptos, GeNeuro, Sanofi Genzyme, Merck, Novartis, Octapharma, Teva Pharmaceuticals, MedImmune, and Roche, fees for serving on advisory boards from Biogen Idec, Sanofi Genzyme, Merck, Novartis Pharmaceuticals, Octapharma, Teva Pharmaceuticals, and Roche, and lecture fees from Biogen, Sanofi Genzyme, Merck, Novartis Pharmaceuticals, Octapharma, Teva Pharmaceuticals, MedImmune, and Roche. MS served on the scientific advisory and/or received speaker honoraria or travel funding from UCB, Biogen Idec, Grifols, Genzyme, Kedrion, Roche, Merck, Novartis, Octapharma, Sanofi-Aventis, TEVA, and Bayer outside the submitted work. The remaining authors declare that the research was conducted in the absence of any commercial or financial relationships that could be construed as a potential conflict of interest.
