# Supplementary material for: Corneal Confocal Microscopy Demonstrates Corneal Nerve Loss in Patients With Trigeminal Neuralgia
Source: Front Neurol. 2020 Jul 24;11:661. doi: 10.3389/fneur.2020.00661 (PMC7393442; doi:10.3389/fneur.2020.00661)
Supplement: Supplementary file 1 [file Data_Sheet_1.pdf]

Supplementary information

Table S1 CCM findings in the ipsilateral and contralateral cornea of patients with TN presented as mean, standard deviation and P-values (P<0.05 considered statistically significant, n.s.- no significant difference).

	**Ipsilateral TN (n=11)**	**Contralateral TN (n=11)**	**P-value**
CNFD (no./mm^2)	22.41 ± 5.69	21.70 ± 7.81	n.s.
CNBD (no./mm^2)	27.89 ± 19.08	27.41 ± 16.63	n.s.
CNFL (mm/mm^2)	13.61 ± 3.58	12.98 ± 4.06	n.s.
CTBD (no./mm^2)	43.09 ± 29.76	40.02 ± 22.76	n.s.
CNFA (μm^2)	5.74 ± 2.12	5.25 ± 1.69	n.s.
CNFW (μm)	19.80 ± 0.51	19.86 ± 0.69	n.s.
DC (F) (no./mm^2)	7.31 ± 7.56	5.37 ± 5.33	n.s.
NC (F) (no./mm^2)	4.14 ± 3.22	2.99 ± 2.58	n.s.
DC (P) (no./mm^2)	15.24 ± 14.01	14.27 ± 11.19	n.s.
NC (P) (no./mm^2)	16.03 ± 17.31	12.68 ± 10.60	n.s.

Table S2 Ipsilateral CCM parameters in TN patients with (n= 4) and without (n= 7) clinical V1 involvement presented as mean, standard deviation and P-values (P<0.05 considered statistically significant, n.s.- no significant difference).

	**TN with V1 involvement (n=4)**	**TN without V1 involvement (n=7)**	**P-value**
CNFD (no./mm^2)	20.33 ± 4.07	23.59 ± 6.42	n.s.
CNBD (no./mm^2)	26.72 ± 14.92	28.56 ± 22.22	n.s.
CNFL (mm/mm^2)	12.62 ± 2.07	14.17 ± 4.26	n.s.
CTBD (no./mm^2)	41.73 ± 20.45	43.87 ± 35.57	n.s.
CNFA (μm^2)	5.53 ± 1.49	5.86 ± 2.52	n.s.
CNFW (μm)	20.03 ± 0.46	19.67 ± 0.52	n.s.
DC (F) (no./mm^2)	8.24 ± 7.97	6.78 ± 7.91	n.s.
NC (F) (no./mm^2)	5.09 ± 4.43	3.60 ± 2.55	n.s.
DC (P) (no./mm^2)	24.46 ± 19.35	9.97 ± 7.14	n.s.
NC (P) (no./mm^2)	24.95 ± 20.72	10.93 ± 14.19	n.s.

Table S3 Ipsilateral CCM findings in TN patients with (n= 6) and without (n= 5) vessel nerve conflict presented as mean, standard deviation and P-values (P<0.05 considered statistically significant, n.s.- no significant difference).

	TN with vessel-nerve conflict (n=6)	TN without vessel-nerve conflict (n=5)	P-value
CNFD (no./mm^2)	23.12 ± 3.00	21.55 ± 8.25	n.s.
CNBD (no./mm^2)	26.93 ± 14.23	29.06 ± 25.57	n.s.
CNFL (mm/mm^2)	13.24 ± 2.49	14.05 ± 4.88	n.s.
CTBD (no./mm^2)	38.85 ± 20.28	48.18 ± 40.50	n.s.
CNFA (μm^2)	5.42 ± 1.68	6.12 ± 2.71	n.s.
CNFW (μm)	20.03 ± 0.45	19.52 ± 0.46	n.s.
DC (F) (no./mm^2)	4.68 ± 6.78	10.46 ± 7.91	n.s.
NC (F) (no./mm^2)	2.91 ± 3.57	5.62 ± 2.21	n.s.
DC (P) (no./mm^2)	12.92 ± 13.77	18.02 ± 15.37	n.s.
NC (P) (no./mm^2)	12.27 ± 13.22	20.54 ± 22.00	n.s.
